# Cultural adaptation and validation of the Arabic short version of the Iconographical Falls Efficacy Scale (Icon-FES): Assessing concern about falling among older adults

**DOI:** 10.1007/s40520-025-02949-1

**Published:** 2025-02-13

**Authors:** Maha M. Almarwani, Worood M. Alharbi, Walid A. Alkeridy

**Affiliations:** 1 Department of Health Rehabilitation Sciences, College of Applied Medical Sciences, King Saud University, 11433 Riyadh, Saudi Arabia; 2 Department of Medicine, College of Medicine, King Saud University, Riyadh, Saudi Arabia; 3 General Administration of Home Health Care, Therapeutic Affairs Deputyship, Riyadh, Saudi Arabia; 4 Department of Medicine, Geriatric Division, University of British Columbia, Vancouver, BC Canada

**Keywords:** Aging, Concern about falling, Fall efficacy, Fall prevention, Psychometric validation, Reliability, Translation, Older adults, Arabic

## Abstract

**Background:**

Concern about falling is a significant issue among older adults, affecting their quality of life and functional independence. Culturally adapted and validated assessment tools are essential for accurately evaluating concern about falling. This study aimed to translate, cross-culturally adapt, and validate the Arabic short version of the Iconographical Falls Efficacy Scale (Icon-FES).

**Methods:**

The translation and cultural adaptation process followed established guidelines. Structural validity was assessed using exploratory factor analysis (EFA). Internal consistency reliability, test–retest reliability, convergent validity, and known-groups validity of the scale were evaluated.

**Results:**

A total of 123 community-dwelling older adults (mean age 69.54 ± 3.48 years; 53.7% male) participated. The Arabic short version of Icon-FES demonstrated strong structural validity, with EFA supporting a unidimensional structure accounting for 73.47% of the variance. It exhibited high internal consistency (Cronbach’s *α* = 0.95) and excellent test–retest reliability (ICC = 0.97). Convergent validity was confirmed through significant correlations with the Arabic Falls Efficacy Scale-International (FES-I; *r*_*s*_ = 0.73, *p* < 0.001), Single Leg Stance (SLS; *r*_*s*_ =  − 0.34, *p* < 0.001), and Five Times Sit-to-Stand Test (5TSTS; *r*_s_ = 0.44, *p* < 0.001). Known-groups validity showed higher scores in females, those with lower education, and a history of falls.

**Conclusions:**

The Arabic short version of Icon-FES is a reliable and valid tool for assessing concern about falling among community-dwelling older adults. It offers an innovative approach through culturally adapted visual elements that could enhance applicability, enabling accurate assessment and supporting targeted interventions among Arabic-speaking older adults.

## Background

As populations age worldwide, promoting healthy aging has become a growing public priority. The World Health Organization (WHO) defines healthy aging as “the process of developing and maintaining the functional ability that enables well-being in older age” [1]. The United Nations (UN) Decade of Healthy Aging 2021–2030 calls for comprehensive, multisectoral, and inclusive action to promote healthy aging, including improving the availability of tools and guidelines to enhance understanding and address aging-related issues [2].

Aligning with these global commitments, Saudi Vision 2030 emphasizes the importance of maintaining the physical health, psychological well-being, and active social participation of its aging population. This commitment is reflected not only in the goal of ensuring good health and well-being but also in the availability and dissemination of national data on older adults, contributing to international standards on age-related statistics [3]. To effectively implement the 2030 Agenda and promote healthy aging, there is a pressing need for culturally adapted and validated tools that address the unique needs and experiences of older adults. Given the shared linguistic and cultural characteristics across Arabic-speaking populations, developing accessible and standardized assessment tools is essential to support aging-related research and interventions in the region.

Physical and psychological factors often limit the ability of older individuals to stay active and maintain independence, posing a substantial challenge to achieving healthy aging [4, 5]. Concern about falling is a common issue that can lead to reduced physical activity, social withdrawal, and a decline in quality of life [6–8]. Concern about falling is often assessed using tools that rely solely on verbal responses and may not fully capture the nuanced experiences of older adults [9–12]. To address these limitations, the Iconographical Falls Efficacy Scale (Icon-FES) was developed by Delbaere et al. [13], incorporating pictorial depictions of daily activities to provide clear and unambiguous contexts [13]. The visual elements of the Icon-FES enhance assessment by making it more accessible to older adults with low literacy levels or cognitive impairments [14, 15]. The short version of the Icon-FES further streamlines this assessment across 10 daily activities while retaining its visual advantages [13]. It has demonstrated robust psychometric properties in diverse linguistic and cultural settings, including Chinese, Turkish, and Portuguese adaptations [16–18]. Moreover, research suggests that the cultural adaptation process is enhanced by incorporating expressions and images reflective of the target community’s norms and values [19–21].

Despite the high prevalence of falls among older adults in Arab regions, research on concern about falling and related outcomes is limited [22–25]. Recognizing the importance of screening for concern about falling as a critical step in integrating best practices and implementing evidence-based fall prevention interventions, it is essential to have culturally adapted and validated assessment tools. Ensuring the availability of the short version of Icon-FES in Arabic is crucial for accurately assessing concern about falling and improving the quality of life among Arabic-speaking older adults. Therefore, the present study aimed to translate and cross-culturally adapt the short version of Icon-FES into Arabic and evaluate its psychometric properties, including reliability and validity, among community-dwelling older adults.

## Methods

### Design and participants

This cross-sectional study adhered to the guidelines set forth by the Consensus-based Standards for the Selection of Health Measurement Instruments (COSMIN) for the translation, cultural adaptation, and psychometric validation of scales [26]. Participant recruitment was carried out at the King Salman Social Center in Riyadh, Saudi Arabia, from February to June 2023. The study included individuals aged 60 years or older who were able to walk independently, with or without the use of an assistive device. Participants with uncompensated hearing or visual impairments, or those unable to fully understand the study after reviewing the consent form were excluded.

Ethical approval for this research was obtained from the College of Medicine Institutional Review Board at King Saud University (E-23–8155), and all participants provided informed consent prior to participation.

### Sample size estimation

In accordance with the COSMIN reporting guidelines for studies on measurement properties, a minimum sample size of 100 participants is recommended to ensure adequate statistical power for assessing reliability and validity [26]. To account for potential dropouts, we invited more participants than the minimum required.

### Translation and cross-cultural adaptation

The translation and cross-cultural adaptation of the Icon-FES short version into Arabic were conducted with the approval of the scale’s developer [13]. The translation process adhered to established guidelines for cross-cultural adaptation of self-report measures to ensure semantic and conceptual equivalence. This process was guided by both Beaton’s guidelines and the COSMIN methodology, involving key steps such as forward translation, synthesis of translations, backward translation, expert committee review, and pretesting [26, 27].

Two independent forward translations of the English version into Arabic were produced by bilingual translators (one with a medical background, and the other without). The two translations were synthesized into a single reconciled Arabic version by a group of bilingual physical therapists and the translators. Subsequently, two independent back-translations were created by two bilingual translators unfamiliar with the original scale.

An expert committee of 10 multidisciplinary healthcare professionals (geriatricians, physical therapists, occupational therapists, nurses, and health educators) reviewed all translation documents. They assessed semantic and conceptual equivalence with the original scale, as well as clarity using the Content Validity Index (CVI). Each item was rated for relevance on a 4-point scale (1 = not relevant to 4 = highly relevant and clear), with a score of ≤2 indicating irrelevance. Clarity was evaluated using a binary scale (clear or unclear), and feedback was documented.

In addition to the linguistic adaptations, the visual elements of the Icon-FES were culturally tailored to the Middle Eastern cultural context. With Dr. Delbaere’s permission [13], original images were replaced with visuals reflecting Middle Eastern norms and social environments. For example, men’s Western attire was changed to the traditional Thobe and Shemagh (a traditional garment and headscarf), while women’s attire was depicted as the Abaya (a traditional loose overgarment) and headscarf. Facial features were adjusted to represent Middle Eastern appearances, enhancing cultural identification. Environmental contexts within the images were adapted to reflect familiar settings. Outdoor scenes incorporated recognizable landmarks such as mosques, and indoor settings were adjusted to depict typical household interiors and furnishings.

**Fig. 1:**
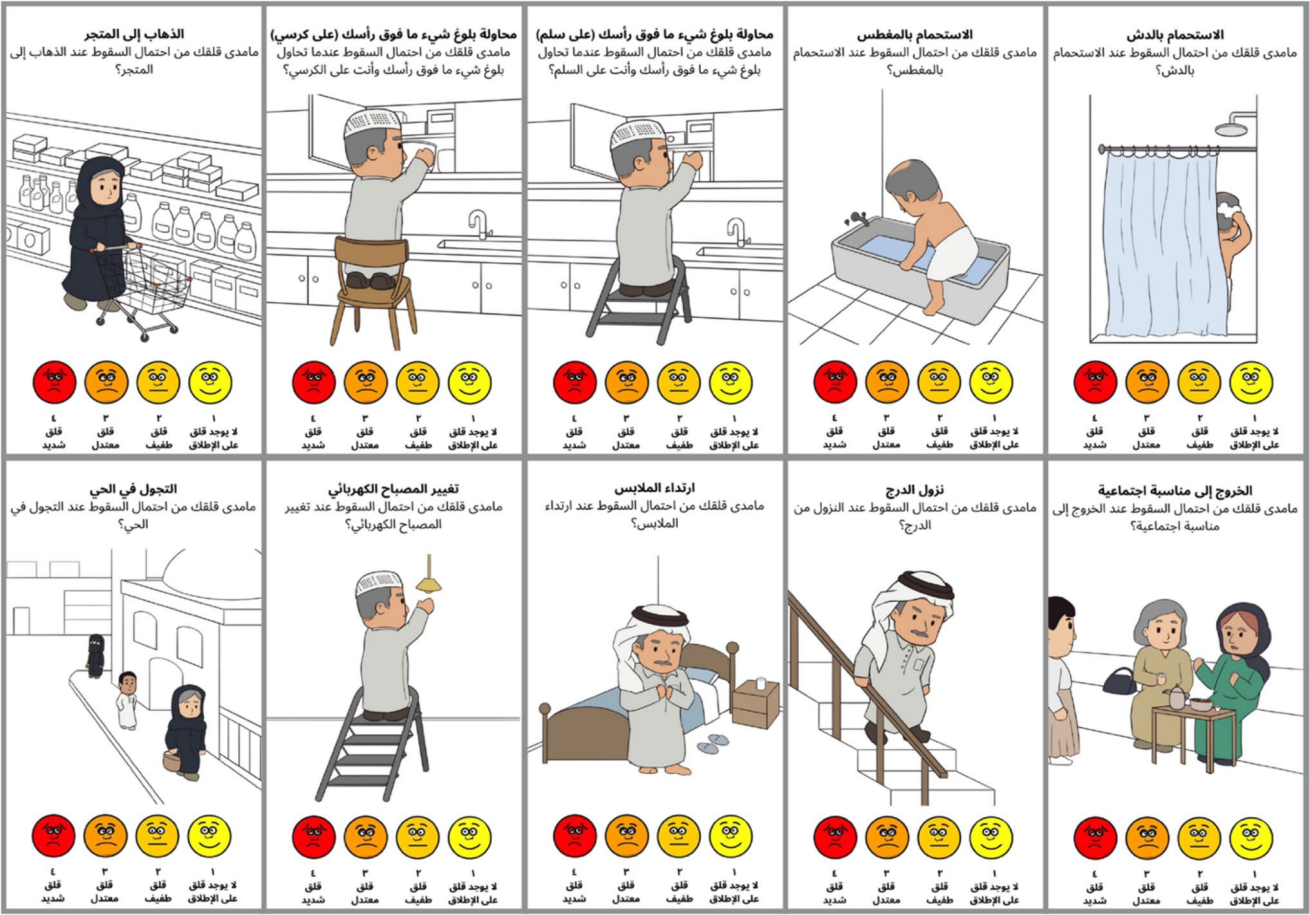
The Arabic short version of Icon-FES

The expert committee reviewed these visual adaptations to ensure they accurately represented the cultural context without altering the conceptual meaning of each item. Following the content validity assessment, the pre-final Arabic version was pretested in a pilot study with 30 community-dwelling older adults to assess the face validity and comprehensibility of the culturally adapted Icon-FES. Feedback from participants was reviewed and discussed by the expert committee, which then incorporated necessary revisions. After this pretesting phase, the final Arabic version of the Icon-FES was produced for psychometric evaluation and large-scale testing (Fig. 1).

### Measures

#### Iconographical Falls Efficacy Scale short version (Icon-FES)

The Icon-FES short version assesses the level of concern about falling across 10 activities of daily living using a combination of pictures and matching short phrases. Participants were instructed to carefully observe each picture and imagine themselves performing the activity. Each item is rated on a 4-point scale, ranging from 1 (not at all concerned) to 4 (very concerned), accompanied by facial expression icons to visually represent the level of concern [13]. The total score for the 10-item Icon-FES ranges from 10 to 40, with scores of 10–18 indicating low concern about falling and scores of 19–40 reflecting high concern [15].

#### Fall history

A fall was defined as unintentionally coming to rest on the ground, floor, or a lower level [28]. In this study, participants were asked whether they had experienced any falls in the past 12 months, and the number of falls was recorded.

#### Falls Efficacy Scale-International (FES-I)

The Arabic version of the Falls Efficacy Scale-International (FES-I) was used to assess fall self-efficacy. This scale measures the level of concern about falling during 16 social and physical activities, ranging from simple to more demanding tasks, regardless of current engagement in these activities. Each item is rated on a 4-point scale (1 = not at all concerned to 4 = very concerned). The total score ranges from 16 to 64, with higher scores indicating greater concern about falls. The Arabic version of the 16-item FES-I has been validated and shown to be reliable among older adults (Cronbach’s *α* = 0.92) [29]. A score of 23 or higher indicates high concern about falling, while a score below 23 indicates low concern [30].

#### Single Leg Stance (SLS)

The Single Leg Stance (SLS) test was used to assess static balance [31]. Participants were instructed to stand on their preferred leg for up to 30 s without external support. The non-stance leg was lifted behind the body, with the knee bent at approximately 90° and the hip in a neutral position. Participants were allowed to position their arms as needed, but the non-stance foot could not touch the ground once timing began. The assessor stood nearby for safety but did not provide support. Timing started when the participant appeared stable and ended after 30 s or if the participant touched down with the non-stance foot or required external support. The best of the two trials was used for statistical analysis. The SLS has been shown to have excellent reliability in older adults [32].

#### Five Times Sit-to-Stand Test (5TSTS)

The Five Times Sit-to-Stand Test (5TSTS) assesses functional ability in performing sit-to-stand transfers [33]. Participants were instructed to rise and sit five times consecutively, as quickly as possible, without using their arms, from a standard chair with an average seat height of 45 cm. The assessor ensured full standing (full extension) and sitting (touching the chair) between repetitions. Timing started when the participant’s back was against the chair and ended upon completion of the fifth repetition. If the participant used their arms or failed to stand or sit properly, the trial was repeated. The 5TSTS has been shown to reliably identify community-dwelling older adults at risk of falls [34, 35].

### Statistical analysis

All statistical analyses were conducted using SPSS® Statistics version 26 (IBM Corp., Armonk, NY, USA). Descriptive statistics were computed to summarize the demographic and clinical characteristics of the participants. The distribution of each variable was assessed using the Shapiro–Wilk test for normality.

The internal consistency of the Arabic short version of Icon-FES was assessed using Cronbach’s alpha (*α*) coefficient, with a value of ≥0.70 considered acceptable [36]. Test–retest reliability was evaluated using intraclass correlation coefficients (ICCs) with a two-way random-effects model for absolute agreement [ICC (2,1)], along with 95% confidence intervals (CIs), based on scores obtained at baseline and at the 1-week follow-up. ICC values were interpreted as follows: values between 0.50 and 0.75 indicate moderate reliability; values between 0.75 and 0.90 indicate good reliability; and values above 0.90 indicate excellent reliability [37]. Floor and ceiling effects were assessed by calculating the percentage of participants achieving the lowest (minimum) or highest (maximum) possible scores. A floor or ceiling effect was considered present if more than 15% of participants achieved the lowest or highest possible score, respectively [38].

An exploratory factor analysis (EFA) using principal axis factoring was conducted to assess the structural validity of the Arabic short version of Icon-FES. The suitability of the data for factor analysis was evaluated using the Kaiser–Meyer–Olkin (KMO) measure of sampling adequacy and Bartlett’s Test of Sphericity. A KMO value greater than 0.50 and a significant Bartlett’s test (*p* < 0.05) indicated the appropriateness of the data for factor analysis [39].

Convergent validity was evaluated by examining the correlations between the Arabic short version of Icon-FES scores and related constructs, including measures of concern about falling (FES-I) and physical performance (SLS and 5TSTS). Since all measures were non-normally distributed, non-parametric Spearman’s rank correlation coefficients (*r*_*s*_) were calculated. Correlation coefficients were interpreted based on their magnitude as follows: strong above 0.70, moderate between 0.30 and 0.70, and weak below 0.30 [40].

Known-groups validity was assessed by comparing the Arabic short version of Icon-FES scores across groups defined by demographic variables (gender, education level, and fall history). The Mann–Whitney *U* test was used for group comparisons due to non-normal distributions. Effect sizes (*r*) for the Mann–Whitney *U* tests were calculated using the formula:

$$r=\frac{Z}{\sqrt{N}}$$

where *Z* is the test statistic from the Mann–Whitney *U* test and *N* is the total sample size [41]. Effect sizes were interpreted according to Cohen’s guidelines for correlation coefficients: small effect (*r* = 0.10 to < 0.30), medium effect (*r* = 0.30 to < 0.50), and large effect (*r* ≥ 0.50) [42]. All statistical tests were two-tailed, and a *p*-value less than 0.05 was considered statistically significant.

## Results

### Participants

**Fig. 2:**
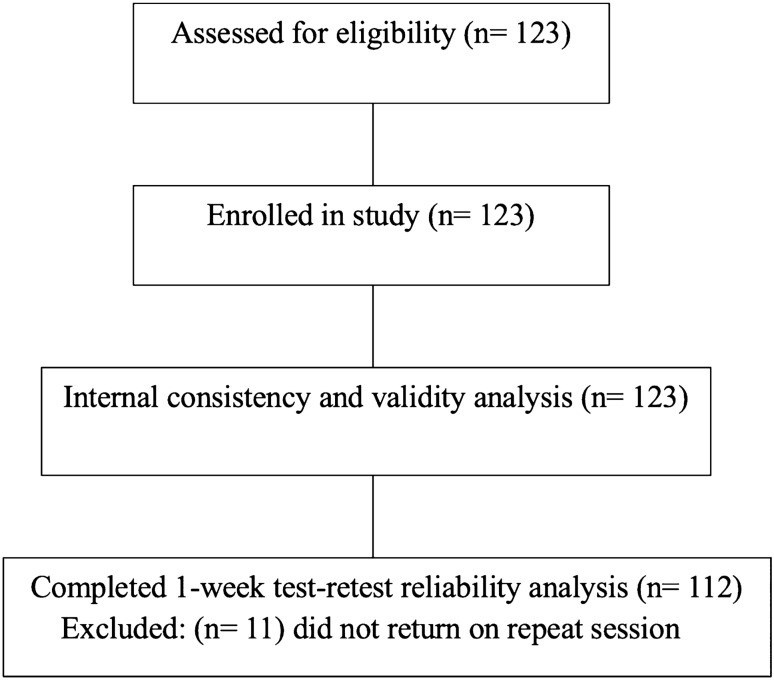
Flow chart of the participants

A total of 123 community-dwelling older adults participated in the study. The mean age of participants was 69.54 years (SD = 3.48), and 53.7% (*n* = 66) were male. Approximately 12.2% (*n* = 15) reported experiencing at least one fall in the previous year. Based on the classifications of both the Falls Efficacy Scale-International (FES-I) and the short version of Icon-FES, the majority of participants indicated low concern about falling. Regarding physical performance, participants demonstrated good balance and mobility, with a mean Single Leg Stance (SLS) time of 25.80 s (SD = 6.27) and a mean Five Times Sit-to-Stand Test (5TSTS) time of 12.26 s (SD = 0.92). Additional participant characteristics are presented in Table 1. A flowchart illustrating participant recruitment is shown in (Fig. 2).

**Table 1:** Characteristics of participants (*n* = 123)

Characteristics	Mean ± *S*D or *n* (%)
Age (years)	69.54 ± 3.48
*Gender*	
Male	66 (53.7%)
Female	57 (46.3%)
*Education level*	
Primary education	34 (27.6%)
Secondary education and higher	89 (72.4%)
*Fall history*^a^	
No	108 (87.8%)
Yes	15 (12.2%)
*Physical performance*	
Single Leg Stance (SLS) (s)	25.80 ± 6.27
Five Times Sit-to-Stand Test (5TSTS) (s)	12.26 ± 0.92
*Concern about falling measures*	
Falls Efficacy Scale-International (FES-I)	21.26 ± 7.23
High concern about falling (scores > 23)	18 (14.6%)
Low concern about falling (scores ≤ 23)	105 (85.4%)
The Arabic short version of Icon-FES	14.82 ± 5.2

FES-I, Fall Efficacy Scale International; Icon-FES, Iconographical Falls Efficacy Scale; SD, Standard deviation

^a^Fall history was assessed by a yes/no question: “Have you fallen in the past 12 months?”

### Translation and cross-cultural adaptation

The translation and cross-cultural adaptation of the Iconographical Falls Efficacy Scale short version (Icon-FES) into Arabic were successfully completed. Feedback from both experts and participants was incorporated to refine the scale further, ensuring both cultural and contextual suitability. The culturally adapted Arabic short version of Icon-FES, as shown in Fig. 1, was well received by participants, indicating high acceptability and cultural relevance.

### Face and content validity

The expert committee’s assessment resulted in high content validity for the Arabic short version of Icon-FES. The Scale Content Validity Index, Average (S-CVI/Ave), was 0.98, and the Scale Content Validity Index, Universal Agreement (S-CVI/UA), was 0.85, indicating a high level of content validity for the scale. Each item’s Content Validity Index (I-CVI) ranged from 0.90 to 1.00, demonstrating that all items were highly relevant.

In the pilot study with 30 community-dwelling older adults (mean age = 68.50 years, SD = 2.71; 56.7% female; 40% with higher education), participants reported no difficulties understanding the culturally adapted items. This supports the scale’s face validity, indicating that it is clear and easily understandable. Feedback confirmed that the content and structure of the Arabic short version of Icon-FES were appropriate, reinforcing its suitability for the target population.

### Structural validity

The KMO measure was 0.90, indicating excellent sampling adequacy. Bartlett’s Test of Sphericity was significant (*χ*^*2*^ = 1647.35, *df* = 45, *p* < 0.001), confirming that the correlation matrix was suitable for factor analysis. The EFA revealed a single-factor solution, supported by the scree plot and eigenvalues. This single factor accounted for 73.47% of the total variance (eigenvalue = 7.347), indicating that the Arabic short version of Icon-FES is a unidimensional measure of concern about falling among older adults. Given the unidimensionality of the scale, no rotation was applied.

Table 2 presents the mean scores, standard deviations, factor loadings, and communalities for each item. All items demonstrated strong associations with the underlying factor, with factor loadings ranging from 0.669 to 0.923 and communalities ranging from 0.447 to 0.853. No cross-loadings were observed, reinforcing the scale’s structural validity.

**Table 2:** Exploratory factor loading of items on the Arabic short version of Icon-FES

Items	Mean ± SD	Loadings	Communalities
1. Going to the shop	1.11 ± 0.37	0.85	0.72
2. Reaching for something above your head (on a chair)	1.97 ± 0.65	0.80	0.63
3. Reaching for something above your head (on a ladder)	2.07 ± 0.72	0.86	0.74
4. Taking a bath	1.59 ± 0.91	0.81	0.65
5. Taking a shower	1.25 ± 0.65	0.92	0.85
6. Walking around in the neighborhood	1.15 ± 0.46	0.92	0.85
7. Changing a lightbulb	2.11 ± 0.46	0.89	0.79
8. Getting dressed	1.06 ± 0.27	0.67	0.45
9. Going down the stairs	1.37 ± 0.27	0.91	0.83
10. Going out to a social event	1.13 ± 0.40	0.92	0.84
**Total variance explained**			73.47%

### Reliability

The internal consistency of the Arabic short version of Icon-FES was excellent, with a Cronbach’s alpha (*α*) of 0.95 (95% CI: 0.94 to 0.96). Corrected item-total correlations ranged from 0.65 to 0.90, indicating strong correlations between individual items and the overall scale score. The Cronbach’s alpha (*α*) remained high (0.94–0.95) when each item was deleted, suggesting that all items contributed meaningfully to the scale’s reliability (Table 3). The test–retest reliability was excellent, with an ICC (2,1) of 0.97 (95% CI: 0.96 to 0.98), based on a test–retest reliability sample of 112 participants who completed the questionnaire at baseline and at a 1-week follow-up. This high level of reliability indicates that the Arabic short version of Icon-FES provides consistent and stable measurements of concern about falling over time. No floor or ceiling effects were found in administrations of the Arabic short Icon-FES score.

**Table 3:** Internal consistency of the Arabic short version of Icon-FES

Items	Corrected item- total correlations	Cronbach’s *α* if item deleted
1. الذهاب إلى التسوق Going to the shop	0.82	0.95
2. محاولة الوصول الى شيء ما أعلى من مستوى رأسك (على كرسي) Reaching for something above your head (on a chair)	0.79	0.95
3. محاولة الوصول الى شيء ما أعلى من مستوى رأسك (على سلم) Reaching for something above your head (on a ladder)	0.86	0.94
4. الاستحمام بالمغطس Taking a bath	0.79	0.95
5. الاستحمام بالدش Taking a shower	0.90	0.94
6. التجول في الحي Walking around in the neighborhood	0.89	0.94
7. استبدال المصباح الكهربائي Changing a lightbulb	0.89	0.94
8. ارتداء الملابس Getting dressed	0.65	0.95
9. النزول من الدرج Going down the stairs	0.90	0.94
10. الخروج إلى مناسبة اجتماعية Going out to a social event	0.88	0.95
**Total Cronbach’s *α***	0.95 (95% CI: 0.94–0.96)	

### Convergent validity

The Arabic short version of Icon-FES demonstrated good convergent validity, as evidenced by significant correlations with established measures related to concern about falling and physical performance. There was a strong positive correlation with the Falls Efficacy Scale-International (FES-I) (*r*_*s*_ = 0.73, 95% CI: 0.60 to 0.83, *p* < 0.001), indicating that higher Icon-FES scores are associated with higher levels of concern about falling as measured by the FES-I. Additionally, the Icon-FES showed a moderate negative correlation with Single Leg Stance (SLS) duration (*r*_*s*_ = −0.34, 95% CI: 0.52 to −0.15, *p* < 0.001), suggesting that higher concern about falling is associated with poorer balance performance. A moderate positive correlation was also observed with the Five Times Sit-to-Stand Test (5TSTS) time (*r*_*s*_ = 0.44, 95% CI: 0.25 to 0.61, *p* < 0.001), indicating that higher concern about falling is associated with slower functional mobility.

### Known-Groups validity

The Arabic short version of Icon-FES demonstrated good known-groups validity by effectively distinguishing between groups expected to differ in concern about falling. Significant differences in Icon-FES scores were found based on gender, educational level, and fall history. Females reported higher concern about falling than males (*p* = 0.026, *r* = 0.21). Participants with primary education reported higher concern about falling than those with secondary education or higher (*p* = 0.005, *r* = 0.25). Additionally, participants with a history of falls showed significantly higher concern about falling than those without (*p* < 0.001, *r* = 0.50) (Table 4).

**Table 4:** Known-Groups validity of the Arabic short version of Icon-FES

**Variable**s	**Group**	***n***	**Mean ± SD**	**Median (IQR)**	***p-*value**^**a**^	**Effect size (*r*)**
Gender	Male	66	13.35 ± 2.96	13 (13–14)	0.026	0.21
	Female	57	16.53 ± 6.65	13 (13–20)		
Educational level	Primary education	34	17.03 ± 6.22	14 (13–21)	0.005	0.25
	Secondary education and higher	89	13.98 ± 4.60	13 (10–15)		
Fall History	No	108	13.40 ± 2.74	13 (13–14)	<0.001	0.50
	Yes	15	25.07 ± 7.38	28 (25–32)		

Abbreviations: *r*, effect size for the Mann–Whitney *U* test; IQR, Interquartile range (Q25–Q75)

^a^ Mann–Whitney *U* test

## Discussion

This study is the first to translate, cross-culturally adapt, and validate the short version of the Icon-FES into Arabic for use among community-dwelling older adults. The findings confirm that the Arabic short version of Icon-FES is a reliable and valid tool for assessing concern about falling among community-dwelling older adults. The incorporation of culturally relevant visuals in the Arabic short version of Icon-FES addresses challenges related to limited literacy skills among Arabic-speaking older adults, which may enhance acceptability and engagement [13]. This innovative approach enables participants to more accurately identify their concern about falling, facilitating targeted interventions to improve their quality of life. Due to historical and social factors, education in Arabic-speaking regions, including Saudi Arabia, became widely accessible and formalized only in the mid-twentieth century, resulting in disparities in educational attainment among older adults, particularly among women and rural populations. Adult literacy programs were developed later to address the educational needs of older generations [43].

Aligned with these adaptation efforts, previous studies have also modified the Icon-FES to enhance cultural relevance in diverse settings. For instance, the Chinese version of the Icon-FES substituted specific items to better reflect common environmental contexts in China, such as replacing “Going down the stairs” with “Using backstairs within a building,” given the prevalence of multistory buildings [16]. Similarly, the Brazilian version made adjustments to two items from the original long version to ensure conceptual and cultural alignment [17]. These adaptations highlight the critical importance of tailoring assessment tools to align with specific cultural and environmental factors of particular populations, thereby enhancing their validity and acceptability. By aligning with these global adaptation efforts, our study reinforces the significance of cultural tailoring in developing reliable and valid assessment tools for concern about falling.

The exploratory factor analysis confirmed that the Arabic short version of Icon-FES has a unidimensional structure, consistent with the original English version [13]. This finding is significant because it suggests that the scale measures a single underlying construct: concern about falling. This allows for straightforward interpretation of the total score. The unidimensionality supports the practical use of a total score, facilitating efficient assessments in clinical and research settings and enhancing the scale’s utility in developing targeted interventions to address fall-related risks.

The Arabic short version of Icon-FES demonstrated high internal consistency (Cronbach’s *α* = 0.95) and excellent test–retest reliability (ICC = 0.97), indicating that the scale is a reliable tool for assessing concern about falling among older adults. The results pertaining to reliability are comparable to those reported for the original English version and other cultural adaptations, including the Chinese, Brazilian, and Turkish versions [13, 16–18]. These consistent psychometric properties across diverse cultural contexts reinforce the cross-cultural applicability of the Icon-FES.

The Arabic short version of Icon-FES exhibited strong convergent validity, evidenced by a significant positive correlation with the Arabic FES-I. This strong correlation suggests that both instruments effectively measure the underlying construct of concern about falling. Additionally, the moderate correlations between the Arabic short version of Icon-FES and physical performance tests further substantiate its construct validity. Specifically, the moderate negative correlation with Single Leg Stance (SLS) duration and the moderate positive correlation with Five Times Sit-to-Stand Test (5TSTS) time indicate that higher levels of concern about falling are associated with poorer balance and slower functional mobility. These findings align with existing literature [44], indicating that concern about falling is associated with functional impairments, and our results using the Arabic short version of the Icon-FES reinforce the importance of a culturally adapted instrument for comprehensively assessing such concerns in relation to physical abilities.

Additionally, the Arabic short version of Icon-FES demonstrated strong known-groups validity by effectively distinguishing between subgroups based on gender, educational level, and fall history. Females reported higher levels of concern about falling compared to males, aligning with previous research indicating that females often experience greater fear due to perceived vulnerability or differences in risk perception [45, 46]. Participants with lower education levels demonstrated higher concern about falling, which may be attributed to reduced health literacy or limited awareness of fall prevention strategies. Furthermore, individuals with a history of falls had significantly higher concern about falling scores, consistent with literature showing that previous falls heighten fear due to increased awareness of fall risk [47]. These findings validate the capability of the Arabic short version of Icon-FES to accurately assess concern about falling across diverse demographic and clinical subgroups, enhancing its utility in both clinical assessments and research settings aimed at developing targeted interventions to reduce fall-related risks among older adults. By providing a valid and reliable Arabic version, our study contributes to the global effort to assess and address concern about falling among older adults. This tool facilitates the identification of individuals who are concerned about falling and enables the implementation of targeted interventions, thereby enhancing fall prevention efforts among Arabic-speaking older adults.

Some limitations of the present study should be acknowledged. Participants were recruited from a district community center and self-selected to participate, which may represent a group of relatively active older adults. This could limit the generalizability of our findings to more vulnerable populations. Therefore, future research should examine the psychometric properties of the Arabic short version of Icon-FES across more diverse older populations including those in clinical settings. Additionally, reliance on self-report measures may introduce biases such as recall bias or social desirability bias. We also recognize the potential limitations posed by the unequal sample sizes of the subgroups in the known-group validity analysis, which suggests that the results should be interpreted with caution. Future studies could address this by investigating measurement invariance with more balanced sample sizes to enhance the generalizability of the findings.

## Conclusion

The Arabic short version of the Iconographical Falls Efficacy Scale (Icon-FES) is a reliable and valid tool for assessing concern about falling among community-dwelling older adults. By incorporating culturally adapted visual elements, this innovative approach could facilitate accurate assessment and enhance acceptability among Arabic-speaking older adults. The availability of the Arabic Icon-FES could enhance the ability of healthcare providers and researchers to identify and address concern about falling, supporting targeted interventions aimed at reducing fall-related risks. Implementing this tool in clinical practice and community programs can contribute to improving the overall well-being and independence of older adults, ultimately influencing fall prevention strategies and promoting healthy aging in line with national and global initiatives.

## Data Availability

The datasets supporting the findings of this study are available upon reasonable request from the corresponding author.
